# Time series study on the effects of daily average temperature on the mortality from respiratory diseases and circulatory diseases: a case study in Mianyang City

**DOI:** 10.1186/s12889-022-13384-6

**Published:** 2022-05-17

**Authors:** Hongju Guo, Peipei Du, Han Zhang, Zihui Zhou, Minyao Zhao, Jie Wang, Xuemei Shi, Jiayi Lin, Yulu Lan, Xiang Xiao, Caiyun Zheng, Xiaofeng Ma, Chengyao Liu, Junjie Zou, Shu Yang, Jiawei Luo, Xixi Feng

**Affiliations:** 1 Mianyang Center for Disease Control and Prevention, Mianyang, China; 2 School of Intelligent Medicine, Chengdu University of Traditional Chinese Medicine, Chengdu, China; 3 School of Public Health, Chengdu Medical College, Chengdu, China; 4 School of Clinical Medicine, Chengdu Medical College, Chengdu, China; 5 School of Public Health, Xiamen University, Xiamen, China; 6 XiangYa School of Public Health, Central South University, Changsha, China; 7 School of Public Health, Sun Yat-sen University, Guangzhou, China; 8 West China Biomedical Big Data Center, West China Clinical Medical College of Sichuan, University, Chengdu, China

**Keywords:** Time series, Daily average temperature, Respiratory diseases, Circulatory diseases, Mortality

## Abstract

**Background:**

Climate change caused by environmental pollution is the most important one of many environmental health hazards currently faced by human beings. In particular, the extreme temperature is an important risk factor for death from respiratory and circulatory diseases. This study aims to explore the meteorological-health effect and find out the vulnerable individuals of extreme temperature events in a less developed city in western China.

**Method:**

We collected the meteorological data and data of death caused by respiratory and circulatory diseases in Mianyang City from 2013 to 2019. The nonlinear distributed lag model and the generalized additive models were combined to study the influence of daily average temperature (DAT) on mortality from respiratory and circulatory diseases in different genders, ages.

**Results:**

The exposure-response curves between DAT and mortality from respiratory and circulatory diseases presented a nonlinear characteristic of the “V” type. Cumulative Relative Risk of 30 days (*CRR*_*30*_) of deaths from respiratory diseases with 4.48 (2.98, 6.73) was higher than that from circulatory diseases with 2.77 (1.96, 3.92) at extremely low temperature, while there was no obvious difference at extremely high temperature. The health effects of low temperatures on the respiratory system of people of all ages and genders were persistent, while that of high temperatures were acute and short-term. The circulatory systems of people aged < 65 years were more susceptible to acute effects of cold temperatures, while the effects were delayed in females and people aged ≥65 years.

**Conclusion:**

Both low and high temperatures increased the risk of mortality from respiratory and circulatory diseases. Cold effects seemed to last longer than heat did.

**Supplementary Information:**

The online version contains supplementary material available at 10.1186/s12889-022-13384-6.

## Background

Since the 1970s, with climate change and environmental pollution, the relationship between meteorological factors and health has gradually become a hot topic of concern to researchers and the general public [1]. Nevertheless, studies on meteorological-health effects started late in China and, have so far been concentrated mainly in developed regions such as coastal cities and provincial capitals rather than those less developed medium- or small-sized cities in western regions [2, 3]. Due to higher air conditioning penetration rate in economically developed regions, studies conducted in these areas may underestimate the intensity of thermal effect [4–6]. The research on cold effect is more suitable for the less developed areas in south China where heating is insufficient and air conditioning penetration is low in winter.

In this study, both meteorological factors and cause-of-death data were collected in Mianyang City, which is a medium-sized and less-developed city in southwest China, with an area of 20,248.4km^2^, a population of 5.31million, and a GDP per capita of $7609. Respiratory disease and circulatory disease ranked second and third respectively among all cause-of-death in China [7]. In 2016, the top three causes of death in Mianyang were circulatory diseases, tumors and respiratory diseases, accounting for 30.79, 24.96 and 23.50%, respectively. The aim of this study is to explore the correlation strength and correlation pattern between meteorological factors, especially extreme weather events and mortality from respiratory or circulatory diseases, and to analyze how meteorological factors affect human health in less-developed southern regions.

## Methods

### Data sources

In our study, the exposure was temperature, the outcome were respiratory and circulatory disease deaths, and the confounders were gender and age. Mianyang Center for Disease Control and Prevention provided the surveillance data extracted from China’s Disease Surveillance Points system (DSPs) on respiratory disease (ICD-10/J00-J99) and circulatory disease (ICD-10/ I00-I99) deaths from 2013 to 2019. We retrieved the meteorological data from the official websites of Meteorological Bureau of Sichuan Province for the year 2013 to 2019. The mean value of daily temperature was defined as the daily average temperature (DAT), while the maximum and the minimum values of the day’s temperature was defined as the daily maximum temperature (DMaxT) and the daily minimum temperature (DMinT) respectively. The difference between the DMaxT and the DMinT was defined as the daily temperature difference (DTD). All temperatures were measured in degrees Celsius (°C). The ratio of mortality at different exposure intensities was defined as relative risk (*RR*). The temperature threshold corresponding to the lowest *RR* was defined as the Minimum Mortality temperature (MMT). Cumulative *RR* with an n-day lag for different exposure intensities was defined as Cumulative Relative Risk (*CRRn*). The data of resident population at the end of each year were derived from the statistical yearbook of Sichuan Province from 2013 to 2019.

### Data analysis

The meteorological data missing for 7 days (0.27%) were recuperated by the *Last Observation Carried Forward* method. Samples with an uncertain time of death or underlying cause of death were removed from the cause-of-death monitoring data. The total number of people in each subgroup was estimated by multiplying the whole population in each year by the composition ratio of the sixth national census [8].

Descriptive statistical analysis was used to describe the meteorological factors and residential mortality from respiratory diseases and circulatory diseases in Mianyang city from 2013 to 2019, to compare differences in the number of deaths among various subgroups, and to compare differences in cumulative mortality among subgroups of different genders and ages. Additive models were used to decompose the time series into long-term trends, seasonal trends, and stochastic fluctuations, respectively. A distributed lagged nonlinear model (DLNM) [9] between DAT and mortality was developed, and the maximum number of lagged days was set to be 30 days. R software version 3.6.0 was used in data visualization. The effects of cold and heat on health were investigated separately by selecting representative extremely low temperature (− 1 °C) and extremely high temperature (31 °C) respectively, based on the observation of the effects of mean temperature on respiratory disease and circulatory disease mortality, and their lagged effects. The minimum value of the *RR* and the Cumulative *RR* with an n-day lag for different exposure intensities was defined as the Minimum of Relative Risk (*RRmin*) and the Minimum of Cumulative Relative Risk (*CRRmin*).

The additive model was chosen for time series decomposition in this study, which means that a time series consists of three parts: *Y*_*t*_ *= T*_*t*_ *+ S*_*t*_ *+ e*_*t*_. *Y*_*t*_ is the actual observation at time *t*, *T*_*t*_ is the long-term trend at time *t*, *S*_*t*_ is the seasonal trend at time *t*, and *e*_*t*_ is the random fluctuations at time *t* [10].

We applied a standard time-series quasi-Poisson regression to derive estimates of temperature mortality associations, reported as *RR*. The regression included a natural cubic spline of time with 7 degrees of freedom per year to control for seasonal and long-term trends, and an indicator of day of the week. We modelled the association with temperature using a distributed lag non-linear model. Natural cubic splines were used for the temperature dimension and for the lag dimension, respectively. This class of models can describe complex non-linear and lagged dependencies through the combination of two functions that define the conventional exposure-response association and the additional lagresponse association, respectively [11]. We used the Akaike information criterion (AIC) to determine the optimal number and location of nodes. We tried some special node numbers and node positions, such as the 10th, 75th, and 90th percentiles of temperature distributions. Finally through AIC, we determined to use 2 internal nodes whose positions were fixed at 15 °C and 25 °C. We extended the lag period to 30 days to include the long delay of the effects of cold and hot.

## Results

Table 1 showed the general characteristics of meteorological factors and daily deaths from respiratory and circulatory diseases in Mianyang City from 2013 to 2019. Daily average of 21.48 deaths from respiratory diseases and 30.51 deaths from cardiovascular diseases in Mianyang City. The DAT, DMaxT, DMinT and DTD were 17.63 °C, 21.56 °C, 14.29 °C and 7.28 °C, respectively. The changes of the DAT, DMaxT, DMinT and DTD all showed obvious seasonal patterns (eFig. 1 in the Supplement). As the correlation coefficients between DAT and the next two indicators were 0.981 and 0.978, respectively, this suggested that the three indicators are highly correlated. The modeling revealed that the time series models for the DAT, DMaxT, and DMinT are similar. As the DAT is more representative, we chose it for our analysis. Long-term trends in mortality over time were controlled by time indicator variables, while the DOW is controlled by the DOW variable.

**Table 1:** General characteristics of meteorological factors and daily deaths from respiratory and circulatory diseases in Mianyang City from 2013 to 2019

	mean	std	min	percentile					max
				***p1***	***p10***	***p50***	***p90***	***p99***	
**Meteorological factors**									
DMaxT (°C)	21.56	8.12	1.70	5.80	10.45	22.50	32.00	35.00	36.80
DminT (°C)	14.29	7.19	−3.00	0	4.00	15.00	23.00	25.54	27.00
DAT (°C)	17.63	7.50	1.00	3.16	7.30	18.50	27.00	30.00	31.10
DTD (°C)	7.28	2.98	0	1.00	3.10	7.00	11.00	14.00	16.00
**Deaths from illness (case)**									
**Respiratory diseases**	21.48	8.37	6.00	8.00	12.00	20.00	33.00	46.00	80.00
**Gender**									
Male	12.46	5.31	2.00	4.00	7.00	12.00	19.00	28.00	51.00
Female	10.02	4.29	1.00	3.00	5.00	9.00	16.00	23.00	30.00
**Age**									
< 65 yrs	3.51	1.75	1.00	1.00	1.00	3.00	6.00	9.00	13.00
≥ 65 yrs	18.98	7.72	4.00	7.00	10.00	18.00	29.50	42.00	71.00
**Circulatory diseases**	30.51	9.25	9.00	13.00	20.00	29.00	43.00	56.00	73.00
**Gender**									
Male	16.49	5.61	3.00	6.00	10.00	16.00	24.00	32.00	44.00
Female	14.01	5.21	2.00	5.00	8.00	13.00	21.00	29.00	41.00
**Age**									
< 65 yrs	7.32	2.81	1.00	2.00	4.00	7.00	11.00	15.00	21.00
≥ 65 yrs	24.18	8.13	6.00	10.00	15.00	23.00	35.00	47.45	64.00

Abbreviations: *P*^*1*^, *P*^*10*^, *P*^*50*^, *P*^*90*^ and *P*^*99*^ are the 1th, 10th, 50th, 90th and 99th percentiles, respectively; Min, the minimum value; Max, the maximum value

Long-term trends showed a downward trend in DAT (Y = 17.80314-0.00015X, *P* < 0.001) (eFig. 1a). Long-term and seasonal trends in both respiratory (Y = 24.41671 + 0.00510X, *P* < 0.001) (eFig. 2a) and circulatory (Y = 19.06098 + 0.00232X, *P* < 0.001) (eFig. 2b) diseases death series showed an upward trend from 2013 to 2019.

eFigure 3 in the Supplement showed the exposure-response curves between DAT and mortality from respiratory diseases and circulatory diseases presented a nonlinear characteristic of “V” type. The intensity of the impact of high temperature on the daily deaths of residents from respiratory and circulatory diseases is mainly short-term and immediate effect, and is strongest on the day of high temperature. The influence intensity of low temperature on death lasts longer, and the impact on death of respiratory diseases is stronger than that of circulatory diseases.

**Fig. 1:**
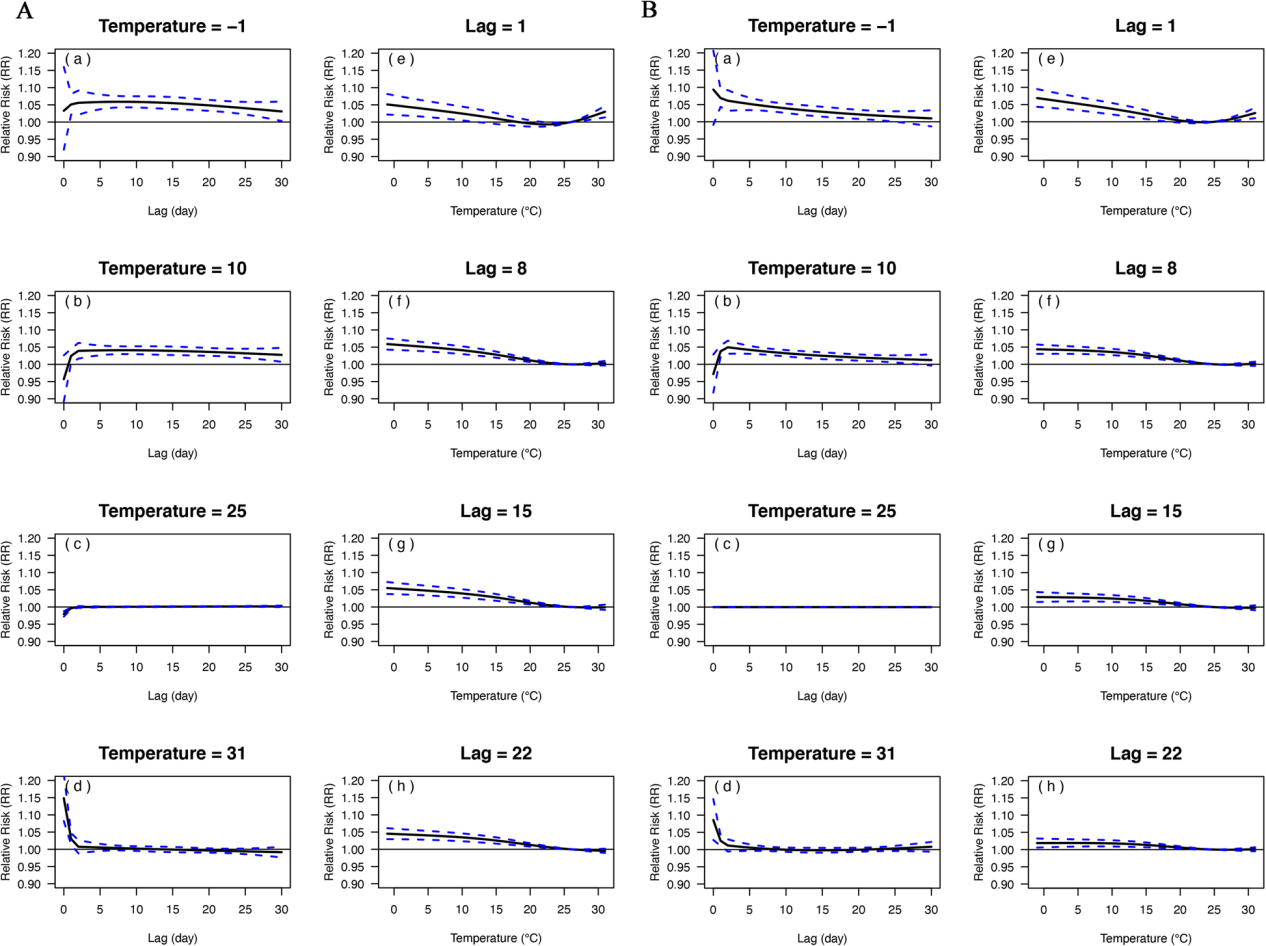
Exposure-response relationships between DAT and mortality from respiratory diseases **A** and circulatory diseases **B**. The RR curves for different lag days at the selected representative temperatures of −1 °C, 10 °C, 25 °C, and 31 °C, and for different temperatures at lag of 1, 8, 15, and 22 days are showed on the left and right, respectively

Plots showing the exposure-lag-response relationships between DAT and mortality from (A) respiratory diseases and (B) circulatory diseases and all diseases were in Fig. 1 and eFigure 4 in the Supplement, respectively. The *RR* curves of − 1 °C and 10 °C both had a longer lag effect on the death from respiratory and circulatory disease, and that this effect diminished with increasing lag days. However, the *RR* curves for respiratory disease deaths were higher and declined more slowly relative to circulatory disease. The *RR* curve of 25 °C is flatter than the other curves as this temperature is close to the MMT. The main difference of the *RR* curve between 31 °C and − 1 °C is the intensity and lag period of the *RR*. The extreme low temperature (− 1 °C) had a more persistent effect than the extreme high temperature (31 °C) on both two diseases.

The overall MMT of deaths from respiratory diseases was 26 °C (Table 2). Cumulative Relative Risk of 30 days (*CRR*_*30*_) became statistically significant when the temperature was lower than 23 °C, and peaked at 4.48 (2.98, 6.73) at the temperature of − 1 °C.

**Table 2:** *CRR*_*30*_ of deaths from respiratory diseases and circulatory diseases at extreme temperature

	Deaths from respiratory diseases			Deaths from circulatory diseases		
	Extremely low temperature	Extremely high temperature	MMT (°C)	Extremely low temperature	extremely high temperature	MMT (°C)
**Overall**	4.48 (2.98, 6.73)*	1.15 (1.00, 1.32)*	26	2.77 (1.96, 3.92)*	1.19 (1.05, 1.36)*	25
**Gender**						
Male	4.67 (2.75, 7.92)*	1.05 (0.90, 1.22)	27	2.08 (1.30, 3.32)*	1.15 (0.99, 1.34)	26
Female	3.92 (2.20, 6.99)*	1.28 (1.02, 1.62)*	25	3.79 (2.31, 6.23)*	1.23 (1.02, 1.48)*	25
**Age**						
< 65 yrs	2.94 (1.21, 7.13)*	1.06 (0.78, 1.45)	26	2.56 (1.30, 5.04)*	1.08 (0.86, 1.35)	26
≥ 65 yrs	4.58 (2.97, 7.05)*	1.16 (1.01, 1.35)*	26	2.77 (1.88, 4.07)*	1.23 (1.06, 1.42)*	25

“*” *P* < 0.05

*CRRs* for extremely low and high temperatures became statistically significant at a lag of 2 days and 1 day, the peak appeared at a lag of 30d and 11d, and lost statistical significance at 30d and 29d, respectively. *CRRs* increased gradually with the increase of lag days at extremely low temperature, and first increased and then decreased at extremely high temperature (eTable 1 in Supplement). The RR for extremely low temperature remained statistically significant from a lag of 1 day to 29 days, and gradually decreased after reaching the peak at a lag of 2 days. The *RR* for extremely high temperature peaked and became statistically significant on the day of high temperature, and ceased to be statistically significant by a lag of 2 days.

**Fig. 2:**
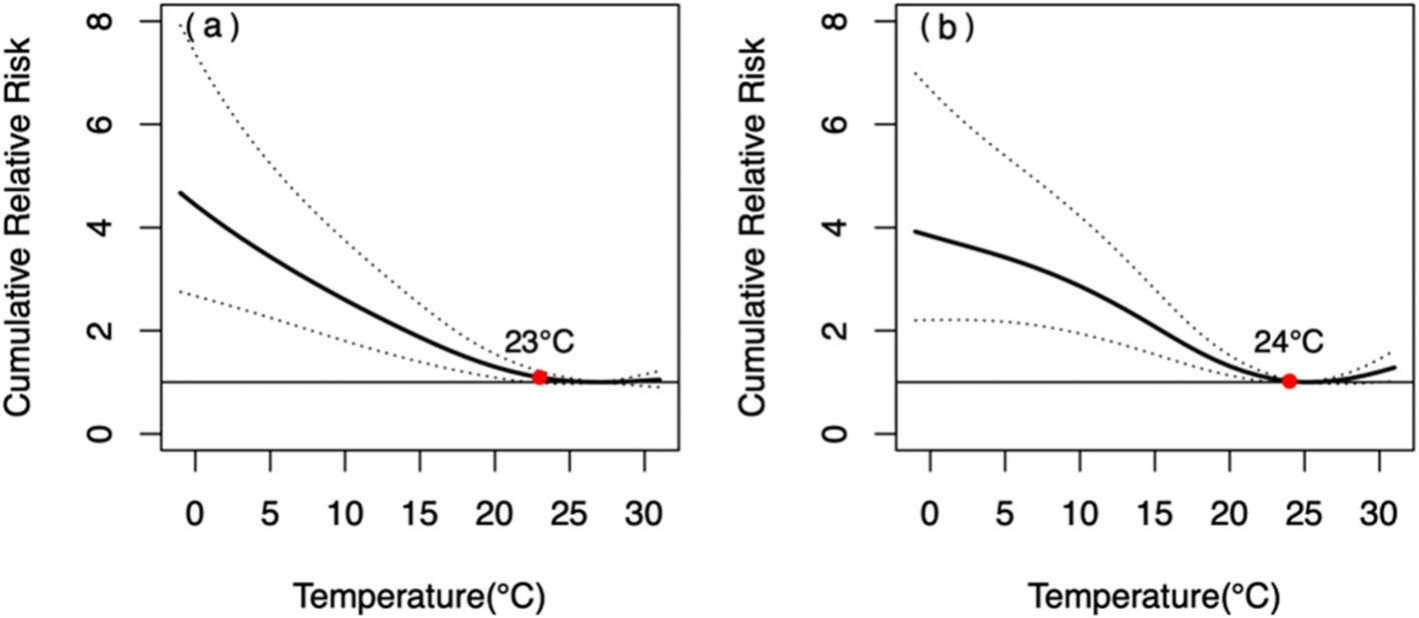
The relationship between DAT and *CRR*_*30*_ of mortality from respiratory diseases in different genders. **a** male, **b** female. The red points indicate that *CRR*_*30*_ is not statistically significant since this temperature

**Fig. 3:**
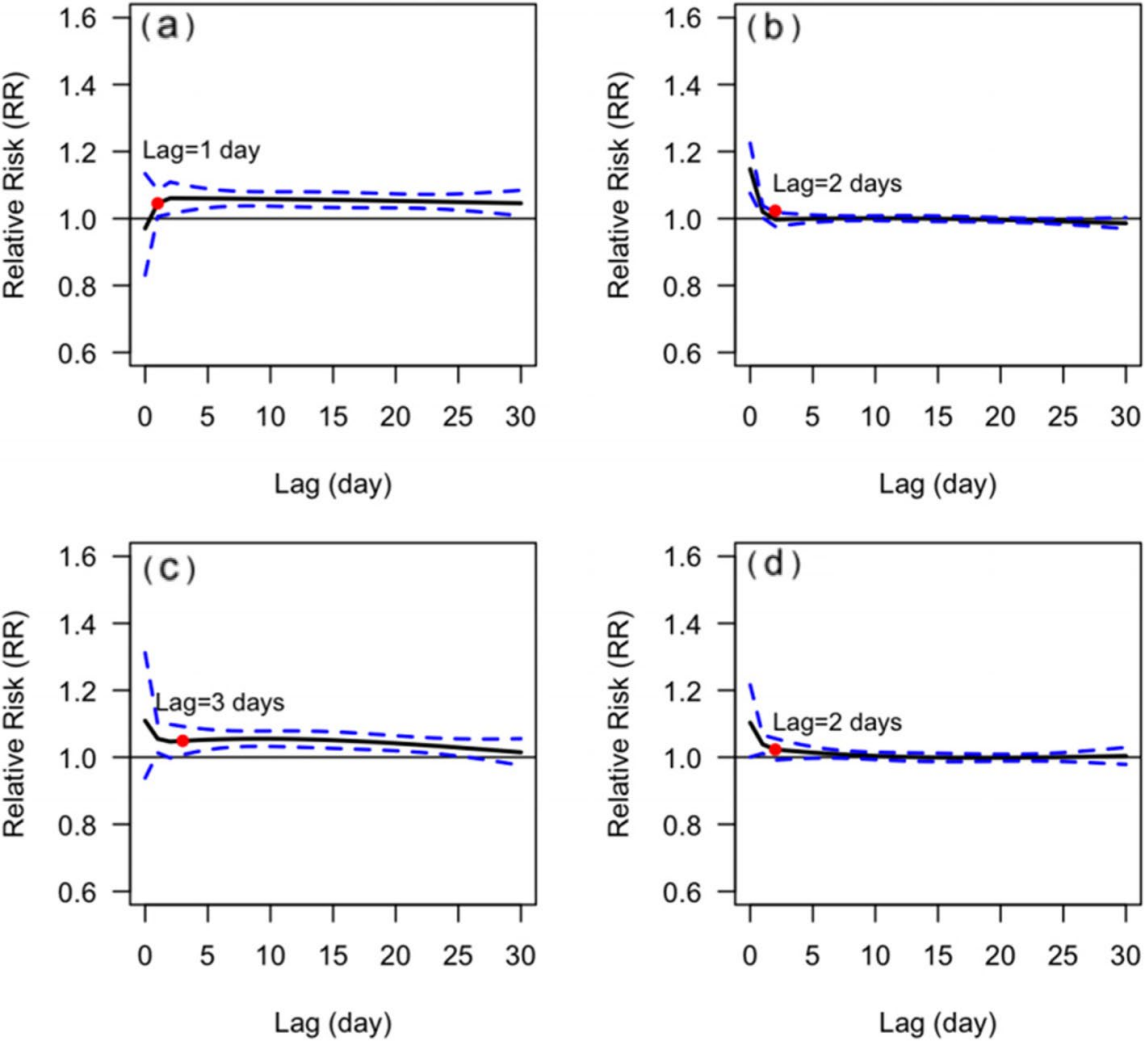
Lagged effects of extremely high and low temperatures on mortality from respiratory disease in different genders. **a** lagged effects of extremely low temperatures on male, **b** lagged effects of extremely high temperatures on the male, **c** lagged effects of extremely low temperatures on the female, and **d** lagged effects of extremely high temperatures on the female. The red points indicate whether the RR is statistically significant since this lag of days

The *CRR*_*30*_ of the male was not statistically significant in the temperature range of 23-31 °C, while the *CRR*_*30*_ of the female was statistically significant when the temperature dropped below 24 °C. Nevertheless, there was no statistically significant gender difference in *CRR*_*30*_ at extreme low or high temperatures, and *p*-value was 0.662 and 0.152, respectively (Fig. 2). The effect of extremely low temperature on mortality from respiratory diseases in male was delayed and lasted for a long time, remaining at high levels until a lag of 30 days, whereas for female, it was acute, reaching a peak on the day of extremely low temperature, then reaching a platform and starting to decline with a lag of about 10 days. In both male and female, the peak *RR* of extremely high temperature occurs on the day when the extremely high temperature occurs and then lost its statistical significance from a lag of 2 days (Fig. 3).

**Fig. 4:**
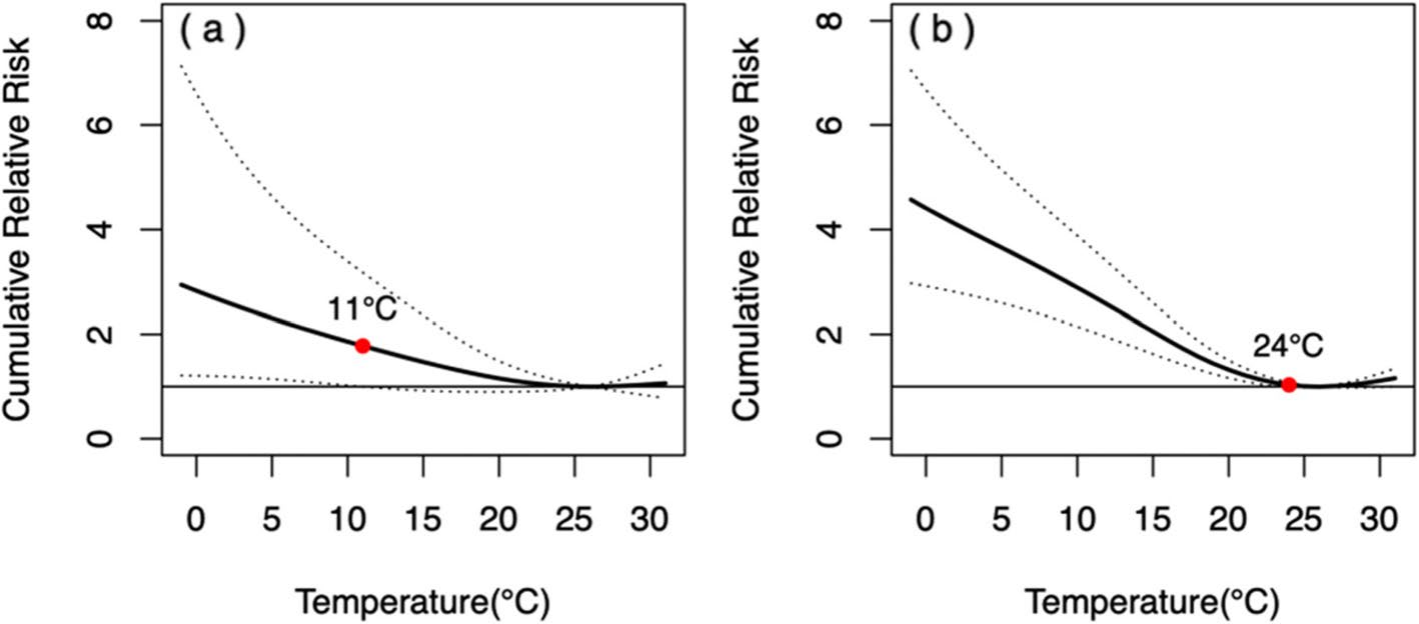
The relationship between DAT and *CRR*_*30*_ of mortality from respiratory diseases in different age group. **a** < 65 yrs., **b** ≥ 65 yrs. The red points indicate that *CRR*_*30*_ is not statistically significant since this temperature

**Fig. 5:**
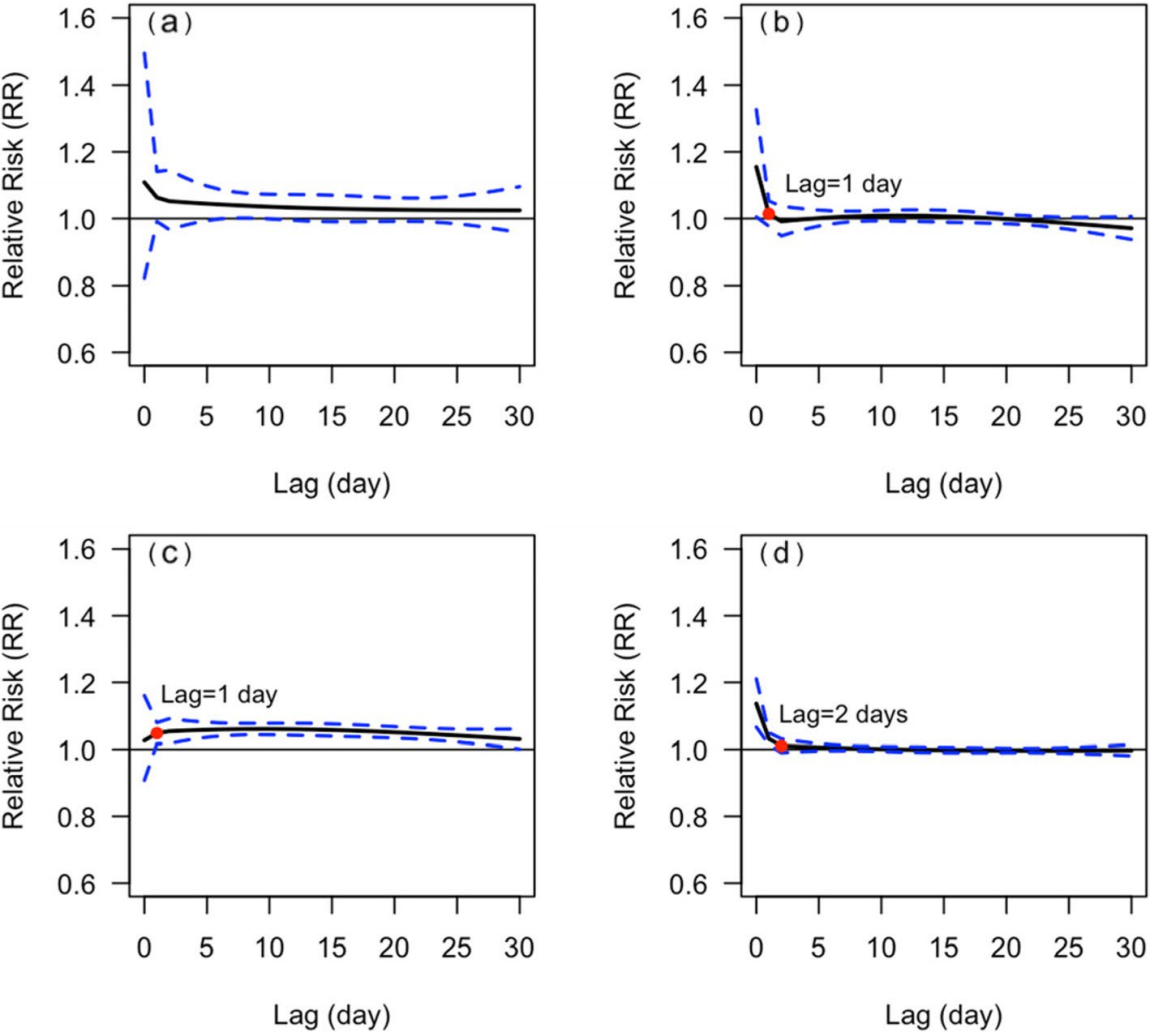
Lagged effects of extremely high and low temperatures on mortality from respiratory diseases in different age groups. **a** lagged effects of extremely low temperatures on people aged < 65 yrs., **b** lagged effects of extremely high temperatures on people aged < 65 yrs., **c** lagged effects of extremely low temperatures on people aged ≥65 yrs., **d** lagged effects of extremely high temperatures on people aged ≥65 yrs. The red points indicate whether the RR is statistically significant since this lag of days

Our study showed that the influence of extreme temperature on mortality from respiratory diseases varied with age. The *CRR*_*30*_ of people aged < 65 yrs. and > 65 yrs. was statistically significant when the temperature dropped below 11°Cand 24 °C, respectively. The influence intensity of extremely low temperature on people aged ≥65 yrs. was slightly higher than that of people aged < 65 yrs. There was no significant difference in CRR30 between different age groups at both extremely low (*P* = 0.379) and extremely high (*P* = 0.618) temperatures (Fig. 4). The effect of extremely low temperatures was acute and declined more rapidly for those < 65 years, but was long-term with a more pronounced lag for those ≥65 years and declined slowly after reaching a peak at the lag of 10 days (Fig. 5).

The overall MMT of circulatory diseases was 25 °C (Table 2). *CRR*_*30*_ became statistically significant when the temperatures was lower than 23 °C, and increase gradually with the decrease of temperature, and peaked at 2.77 (1.96, 3.92) at the temperature of − 1 °C. *CRRs* for extremely low and high temperatures both became statistically significant at a lag of 1 day, and reached peaks of 2.77 (1.96, 3.92) and 1.19 (1.05, 1.36) at a lag of 30d, respectively (eTable 2 in Supplement). The *RR* for extremely high temperature peaked at 1.09 (1.03, 1.15) on the day of high temperature.

**Fig. 6:**
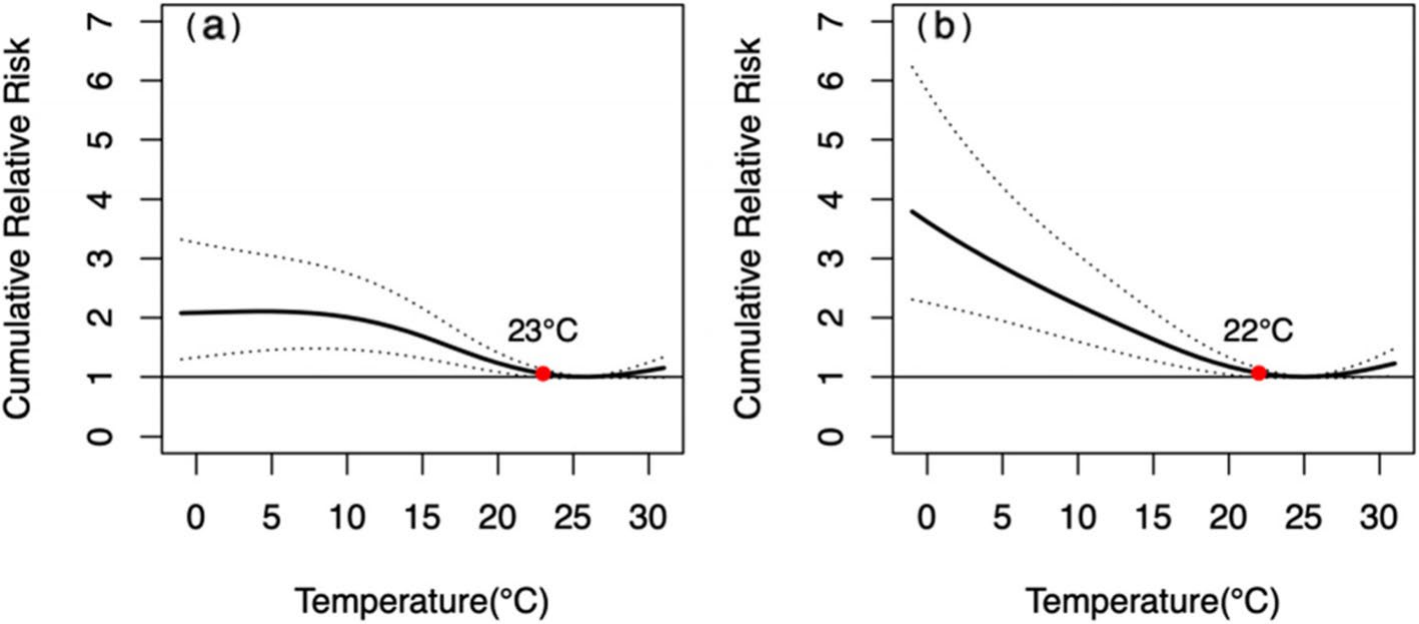
The relationship between DAT and *CRR*_*30*_ of mortality from circulatory diseases in different genders. **a** male, **b** female. The red points indicate that *CRR*_*30*_ is not statistically significant since this temperature

The MMT of male and female was 26 °C and 25 °C respectively (Table 2). The pattern of *CRR*_*30*_ curves from circulatory diseases showed slightly difference between genders (Fig. 6). The influence intensity of extremely low temperature on female was higher than that of male, however, the difference was not statistically significant (*P* = 0.084).

**Fig. 7:**
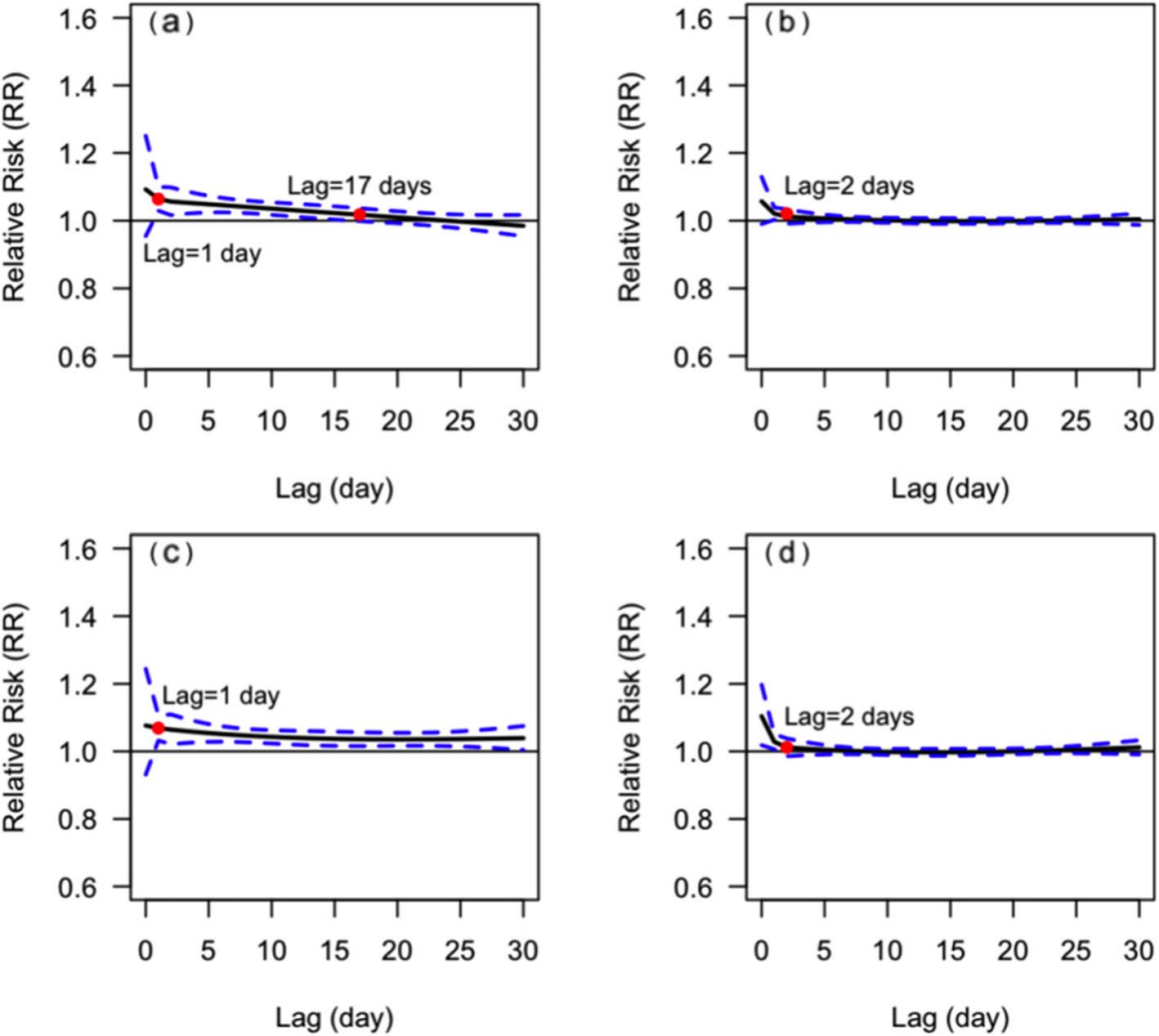
Lagged effects of extremely high and low temperatures on mortality from circulatory diseases in different genders. **a** lagged effects of extremely low temperatures on male, **b** lagged effects of extremely high temperatures on male, **c** lagged effects of extremely low temperatures on female, **d** lagged effects of extremely high temperatures on female. The red points indicate that *RR* is not statistically significant since this lag of days

The peak *RR* of male at extremely low temperature occurred on the lag of 1 day, and gradually decreased until it lost its statistical significance with a lag of 16 days. The RR of male at extremely high temperature was statistically significant only at a lag of 1 day. The *RR* of female at extremely low temperature remained high with a lag of 30 days, but was only statistically significant with a lag of 1 day at extremely high temperature (Fig. 7).

**Fig. 8:**
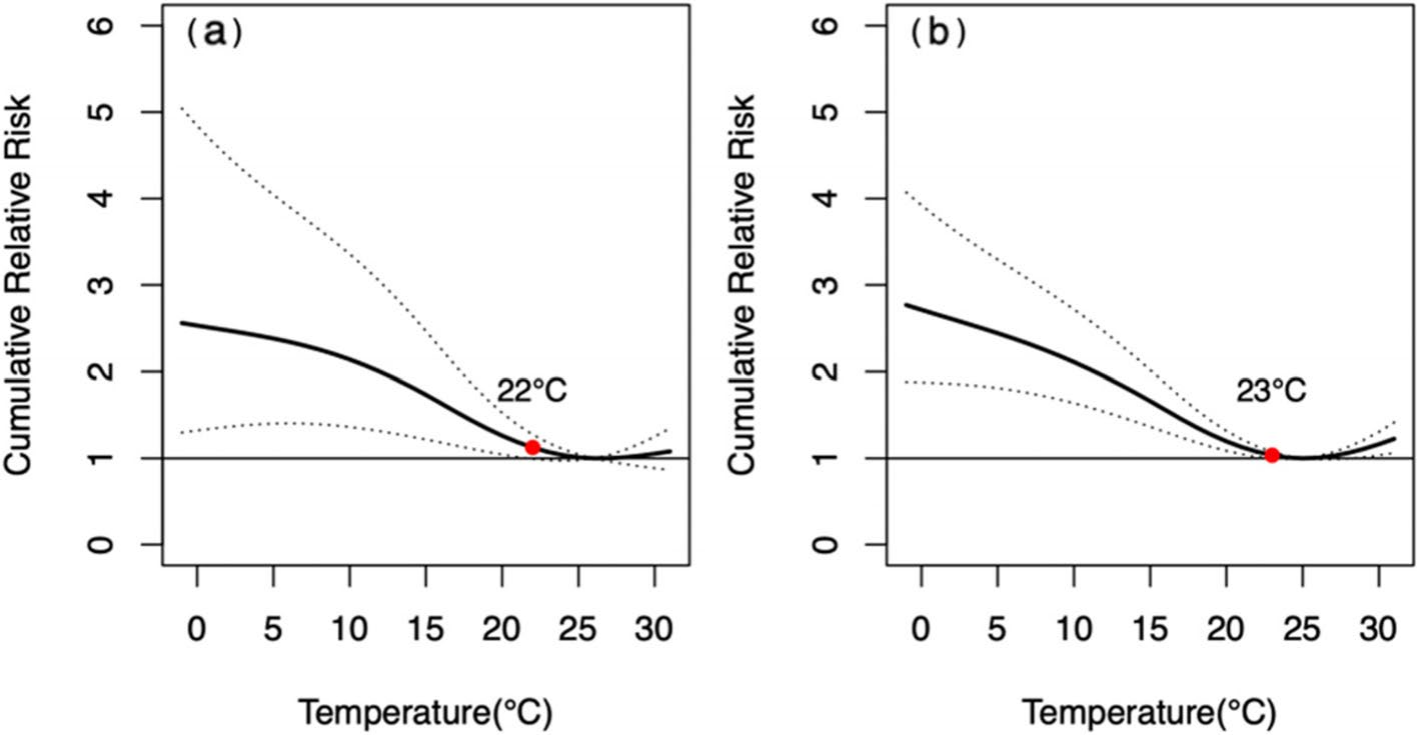
The relationship between DAT and *CRR*_*30*_ of mortality from circulatory diseases in different age groups. **a** people aged < 65 yrs. **b** people aged ≥65 yrs. The red points indicate that *CRR*_*30*_ is not statistically significant since this temperature

**Fig. 9:**
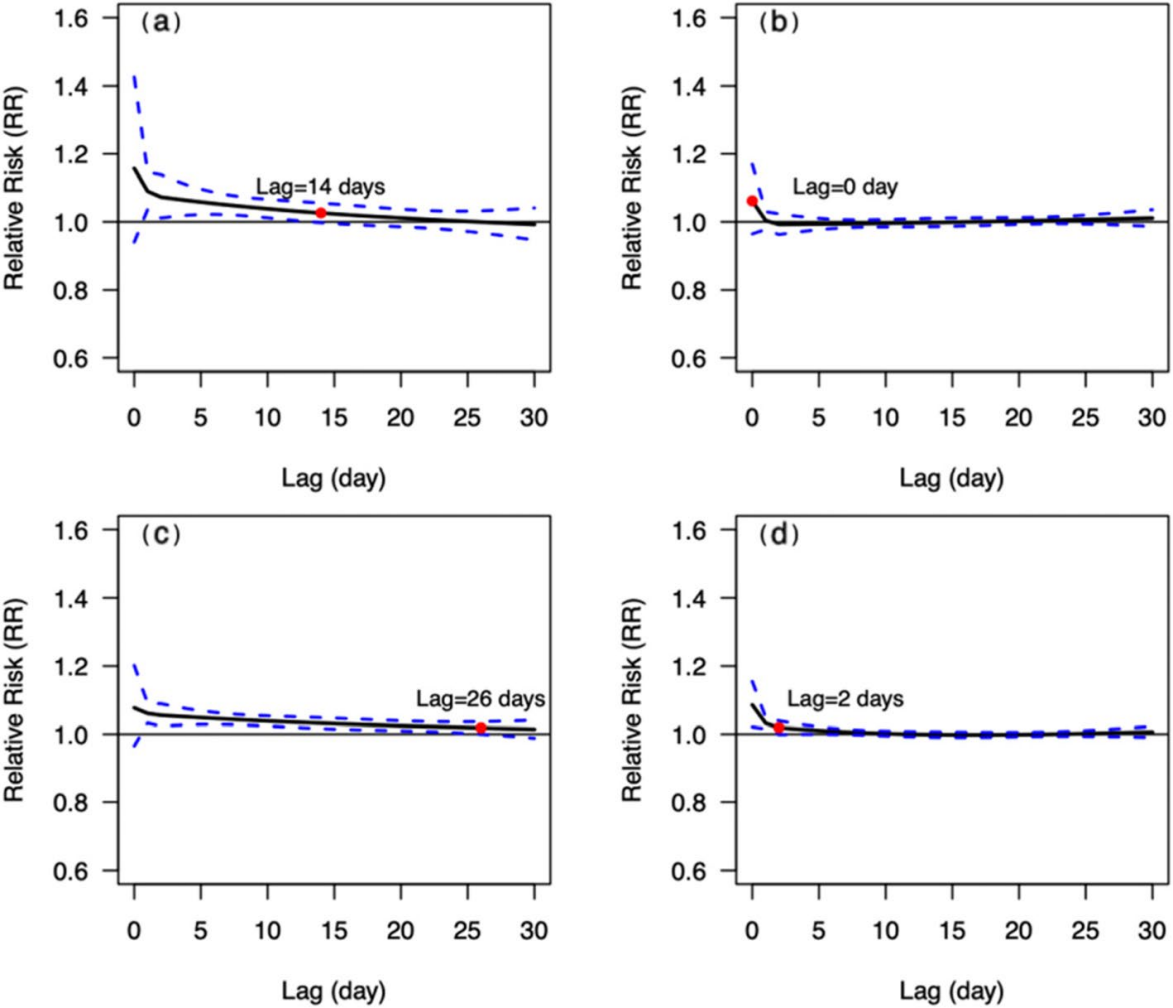
Lagged effects of extremely high and low temperatures on mortality from circulatory diseases in different age groups. **a** lagged effects of extremely low temperatures on people aged < 65 yrs., **b** lagged effects of extremely high temperatures on people aged < 65 yrs., **c** lagged effects of extremely low temperatures on people aged ≥65 yrs., **d** lagged effects of extremely high temperatures on people aged ≥65 yrs. The red points indicate that *RR* is not statistically significant since this lag of days

The MMT of people aged < 65 yrs. and aged ≥65 yrs. were 26 °C and 25 °C respectively (Table 2). As shown in Fig. 8, the patterns of *CRR*_*30*_ curves in both two age groups were similar, and the influence intensity of extremely low temperature in people aged ≥65 yrs. was slightly higher than that in people aged < 65 yrs. *CRR*_*30*_ at extremely high temperature did not show statistically significant (*P* = 0.348) in the two age groups. The extreme cold effects were strongest on the day of extreme low temperature, and declined slowly in both two age groups, losing statistical significance since the lag of 14 and 26 days, respectively (Fig. 9). The effect of extremely high temperature on people aged < 65 yrs. wasn’t statistically significant, while it was only significant on the 0 and 1 day for people aged ≥65 yrs.

## Discussion

In this study, we found that both extremely low and high temperature resulted in an increase in mortality from respiratory diseases and circulatory diseases. The exposure-response curves between DAT and the mortality from respiratory diseases and circulatory diseases presented a nonlinear characteristic of “V” type, which was consistent with recent studies conducted in China [5, 6, 12, 13]. The risks of death from respiratory diseases and circulatory diseases increased when the temperature is below or above 26 °C and 25 °C, respectively. It indicates that there exists a suitable temperature range for human health. The extreme hot effects were acute and short-term, while the extreme cold effects had a certain lag and lasted for a longer period of time, and the cold effect was stronger than the heat effect for both respiratory and circulatory mortality.

Due to the complexity of the climate system, different regions may show different regional climate trends under the background of global warming due to the influence of local natural and geographical conditions. As the study shows, there is a gradual decline in temperature in Mianyang from 2013 to 2019. In developing countries, especially those at middle and high latitudes, the health effects of low temperatures remain of concern due to social and economic conditions, especially given the large and significant lagging effect of low temperatures on respiratory and circulatory diseases. In our study, for both respiratory and circulatory diseases the *CRR*_*30*_ was higher at extremely low temperatures than at extremely high temperatures in Mianyang. The *CRR*_*30*_ of cold effects in Mianyang was higher than that of northern Chinese cities, such as Jinan [14]. The central heating systems in northern cities may account for the difference, as the central heating systems can enhance the adaptability of northern residents to low temperature and reduce the impact of extremely low temperature. Similar to other studies [15], the MMTs in southwest China is about 26 °C, which is higher than that in the northern parts.

The *CRR*_*30*_ of mortality from respiratory diseases at extremely low temperature was higher than that of circulatory diseases, while the *CRR*_*30*_ of respiratory deaths at extremely high temperature was about the same as that of circulatory diseases. These results indicated that the effect of extreme cold on respiratory deaths was stronger than that on circulatory diseases, which is similar to the results of a UK study [16]. The cold effect on the respiratory system lasted for about 30 days, which is consistent with a previous study conducted in Shanghai [17]. However, the intensity of cold effects in Mianyang was stronger than that in Shanghai, which may be caused by good economic conditions, medical accessibility, and higher education level in Shanghai, as we know that respiratory diseases are sensitive to the environment, especially in patients and the elderly [18]. Huang et al. also suggested that people in low-income areas are more vulnerable to high and low temperatures [19]. The common cold and influenza are highly prevalent in winter, and cold temperatures were more likely to increase the risk of cross-infection due to indoor aggregation [20]. This may explain to some extent that low temperature was stronger and delayed longer than high temperature for both diseases. The longer duration of respiratory diseases may be responsible for the hysteresis of the cold effect. The thermal effect on respiratory system lasted no more than 2 days, which is similar to previous studies conducted in cities such as Wuhan, Nanjing, and Shanghai, where the annual average temperature was similar to that of Mianyang [17, 20, 22].

The results of the subgroup analysis suggested that people of all ages and genders should be aware of the ongoing respiratory risks of hypothermia, with those aged < 65 yrs. and female being particularly sensitive on the day of hypothermia, but those aged ≥65 yrs. and male having a delayed response, with the risk of death especially in those aged ≥65 yrs. remaining elevated for 3-10 days after the delay. This might be related to different perceptions of temperature by gender and different physical conditions of people of different ages. Females were generally more sensitive to temperature changes than males, while younger people were usually in better health and have fewer underlying diseases, and were more resistant to cold temperatures.

The duration of the effects of extremely low temperature and extremely high temperature on circulatory system death was 30 days and 1 day, respectively, which was similar to the study conducted in Shanghai. However, our study differed from the study conducted in Shanghai in effect intensity. The *CRR*_*30*_ of thermal effect on circulatory diseases in Mianyang was lower than that in Shanghai. Both the number of statistically significant *CRR*_*30*_ days and the intensity of cold effect in Mianyang were lower than those in Shanghai [17] and Chengdu, another city located in the Sichuan basin [6], indicating that Mianyang residents have a lower susceptibility to circulatory diseases at high and low temperature. Subgroup analysis of mortality from circulatory disease showed that males were almost as susceptible to low temperature as females, and the lasting effect of extreme low temperature on female was longer than that on male, which was related to the physical differences between male and female. Attention should be paid to the ongoing health effects of cold on the circulatory system among all age groups, especially in people aged ≥65 yrs. There was no statistically significant differences in *CRR*_*30*_ across the subgroups, which may be due to the small absolute number of daily deaths, which reduced the test efficiency.

### Limitation

There is some subjectivity in the selection of maximum lag periods, degrees of freedom of exposure and lag dimensions, node locations, smoothing function types, and covariates when building weather-health-related models, which may affect the fitness of the model and the extrapolation of the results. The AIC criterion is a powerful tool to address these issues, thus we have set fixed degrees of freedom for both exposure and lag dimensions by combining previous literature and the AIC criterion in this study. Furthermore, when using the AIC criterion to screen models, care should be taken to combine expertise and consider the interpretability of the results. In addition, tobacco use, health systems and other meteorological factors such as weed speed, relative humidity, and air pressure are also important factors influencing mortality from respiratory and circulatory diseases, and our findings are subject to a degree of bias due to unavailability relevant data. The study used data on the age and gender structure of the population from the sixth national census rather than the actual annual data in Mianyang city, which may result in bias.

## Conclusion

In summary, the residents of Mianyang City were susceptible to both high and low temperatures to a certain extent, but the intensity and lag pattern of the effects of temperature on health varied with gender, age and disease type. The health effects of high temperatures are acute and short-term, while the health effects of low temperatures are chronic with a certain lag. People of all ages and genders should be aware of the ongoing respiratory risks of hypothermia, with those aged < 65 yrs. and female being particularly sensitive on the day of hypothermia, and those over 65 years and male being more concerned about the effects of delay. More attention should be paid to the acute health effects of cold on the circulatory system in people under 65 years, while the ongoing effects should be of greater concern in female and people over 65 years of age.

## Supplementary Information

**Additional file 1.**

## Data Availability

The meteorological data was from the official websites of Meteorological Bureau of Sichuan Province (http://lishi.tianqi.com/mianyang). The surveillance data on respiratory disease (ICD-10/J00-J99) and circulatory disease (ICD-10/ I00-I99) deaths provided by Mianyang Center for Disease Control and Prevention was not available to share.

## Abbreviations

DAT: Daily Average Temperature
CRR30: Cumulative Relative Risk of 30 days
DMaxT: Daily Maximum Temperature
DMinT: Daily Minimum Temperature
DTD: Daily Temperature Difference
RR: Relative risk
MMT: Minimum Mortality Temperature
CRRn: Cumulative Relative Risk of n-day
RRmin: Minimum of Relative Risk
CRRmin: Minimum of Cumulative Relative Risk
DLNM: Distributed Lagged Nonlinear Model
AIC: Akaike Information Criterion
DOW: Day-of-Week
