# Supplementary material for: Time series study on the effects of daily average temperature on the mortality from respiratory diseases and circulatory diseases: a case study in Mianyang City
Source: BMC Public Health. 2022 May 17;22:1001. doi: 10.1186/s12889-022-13384-6 (PMC9115919; doi:10.1186/s12889-022-13384-6)

**Supplementary Online Content**

**eTable 1.** Estimated RR of deaths from respiratory diseases at extreme temperatures

**eTable 2.** Estimated RR of mortality from circulatory diseases at extreme temperatures

**eFigure 1.** Time series decomposition of DAT (a), DMaxT (b), DMinT (c), and DTD (d).

**eFigure 2.** Time series decomposition of deaths from respiratory diseases (a), and circulatory diseases (b).

**eFigure 3.** The 3-D figure of association patterns between DAT and mortality from respiratory diseases (a), circulatory diseases (b), and lag dimensions.

**eFigure 4.** Cross-sectional data on the exposure-response relationships between DAT and mortality from all diseases.

**eTable1: Estimated RR of deaths from respiratory diseases at extreme temperatures**

| **lagging**  **(days)** | **Extremely low temperature** | **Cumulative extremely low temperature** | **Extremely high temperature** | **Cumulative extremely high temperature** |
| --- | --- | --- | --- | --- |
| 0 | 1.03 (0.92, 1.16) | - | 1.15 (1.08, 1.22)* | - |
| 1 | 1.05 (1.02, 1.08)* | 1.09 (0.96, 1.23) | 1.03 (1.01, 1.05)* | 1.18 (1.11, 1.26)* |
| 2 | 1.06 (1.02, 1.09)* | 1.15 (1.02, 1.29)* | 1.01 (0.99, 1.03) | 1.19 (1.12, 1.27)* |
| 3 | 1.06 (1.03, 1.09)* | 1.21 (1.07, 1.37)* | 1.01 (0.99, 1.02) | 1.20 (1.13, 1.28)* |
| 4 | 1.06 (1.03, 1.08)* | 1.28 (1.12, 1.46)* | 1.01 (0.99, 1.02) | 1.21 (1.13, 1.29)* |
| 5 | 1.06 (1.04, 1.08)* | 1.36 (1.18, 1.57)* | 1.01 (1.00, 1.02) | 1.21 (1.13, 1.30)* |
| 6 | 1.06 (1.04, 1.08)* | 1.44 (1.23, 1.67)* | 1.01 (1.00, 1.01) | 1.22 (1.13, 1.31)* |
| 7 | 1.06 (1.04, 1.08)* | 1.52 (1.29, 1.79)* | 1.00 (1.00, 1.01) | 1.22 (1.13, 1.32)* |
| 8 | 1.06 (1.04, 1.08)* | 1.61 (1.35, 1.92)* | 1.00 (1.00, 1.01) | 1.23 (1.13, 1.33)* |
| 9 | 1.06 (1.04, 1.08)* | 1.71 (1.42, 2.05)* | 1.00 (1.00, 1.01) | 1.23 (1.13, 1.34)* |
| 10 | 1.06 (1.04, 1.08)* | 1.80 (1.49, 2.19)* | 1.00 (1.00, 1.01) | 1.23 (1.13, 1.34)* |
| 11 | 1.06 (1.04, 1.08)* | 1.91 (1.56, 2.34)* | 1.00 (0.99, 1.01) | 1.24 (1.13, 1.35)* |
| 12 | 1.06 (1.04, 1.08)* | 2.02 (1.63, 2.50)* | 1.00 (0.99, 1.01) | 1.24 (1.13, 1.35)* |
| 13 | 1.06 (1.04, 1.07)* | 2.13 (1.71, 2.66)* | 1.00 (0.99, 1.01) | 1.24 (1.13, 1.35)* |
| 14 | 1.06 (1.04, 1.07)* | 2.25 (1.79, 2.84)* | 1.00 (0.99, 1.01) | 1.24 (1.13, 1.35)* |
| 15 | 1.06 (1.04, 1.07)* | 2.38 (1.87, 3.03)* | 1.00 (0.99, 1.01) | 1.24 (1.13, 1.36)* |
| 16 | 1.05 (1.04, 1.07)* | 2.51 (1.95, 3.23)* | 1.00 (0.99, 1.01) | 1.23 (1.12, 1.36)* |
| 17 | 1.05 (1.04, 1.07)* | 2.64 (2.03, 3.43)* | 1.00 (0.99, 1.01) | 1.23 (1.12, 1.36)* |
| 18 | 1.05 (1.04, 1.07)* | 2.77 (2.11, 3.65)* | 1.00 (0.99, 1.00) | 1.23 (1.11, 1.36)* |
| 19 | 1.05 (1.03, 1.07)* | 2.91 (2.19, 3.87)* | 1.00 (0.99, 1.00) | 1.22 (1.11, 1.36)* |
| 20 | 1.05 (1.03, 1.07)* | 3.05 (2.27, 4.10)* | 1.00 (0.99, 1.00) | 1.22 (1.10, 1.36)* |
| 21 | 1.05 (1.03, 1.06)* | 3.20 (2.35, 4.34)* | 1.00 (0.99, 1.00) | 1.22 (1.09, 1.35)* |
| 22 | 1.05 (1.03, 1.06)* | 3.34 (2.43, 4.59)* | 1.00 (0.99, 1.00) | 1.21 (1.08, 1.35)* |
| 23 | 1.04 (1.03, 1.06)* | 3.49 (2.51, 4.84)* | 1.00 (0.99, 1.00) | 1.20 (1.08, 1.35)* |
| 24 | 1.04 (1.03, 1.06)* | 3.63 (2.59, 5.09)* | 0.99 (0.99, 1.00) | 1.20 (1.07, 1.34)* |
| 25 | 1.04 (1.02, 1.06)* | 3.78 (2.67, 5.35)* | 0.99 (0.99, 1.00) | 1.19 (1.06, 1.34)* |
| 26 | 1.04 (1.02, 1.06)* | 3.92 (2.74, 5.61)* | 0.99 (0.98, 1.00) | 1.18 (1.05, 1.33)* |
| 27 | 1.04 (1.02, 1.06)* | 4.07 (2.81, 5.88)* | 0.99 (0.98, 1.00) | 1.17 (1.04, 1.33)* |
| 28 | 1.04 (1.01, 1.06)* | 4.21 (2.87, 6.16)* | 0.99 (0.98, 1.01) | 1.17 (1.03, 1.32)* |
| 29 | 1.03 (1.01, 1.06)* | 4.34 (2.93, 6.44)* | 0.99 (0.98, 1.01) | 1.16 (1.01, 1.32)* |
| 30 | 1.03 (1.00, 1.06) | 4.48 (2.98, 6.73)* | 0.99 (0.98, 1.01) | 1.15 (1.00, 1.32) |

“*” *P*<0.05.

**eTable2: Estimated RR of mortality from circulatory diseases at extreme temperatures.**

| **Lagging(days)** | **Extremely low temperature** | **Cumulative extremely low temperature** | **Extremely high temperature** | **Cumulative extremely high temperature** |
| --- | --- | --- | --- | --- |
| 0 | 1.09 (0.99, 1.21) | - | 1.09 (1.03, 1.15)* | - |
| 1 | 1.07 (1.04, 1.10)* | 1.17 (1.05, 1.30)* | 1.03 (1.01, 1.04)* | 1.11 (1.05, 1.18)* |
| 2 | 1.06 (1.03, 1.09)* | 1.24 (1.12, 1.37)* | 1.01 (0.99, 1.03) | 1.13 (1.07, 1.19)* |
| 3 | 1.06 (1.03, 1.08)* | 1.31 (1.18, 1.46)* | 1.01 (1.00, 1.02) | 1.14 (1.07, 1.21)* |
| 4 | 1.06 (1.03, 1.08)* | 1.38 (1.24, 1.55)* | 1.01 (1.00, 1.02) | 1.15 (1.08, 1.22)* |
| 5 | 1.05 (1.03, 1.07)* | 1.46 (1.29, 1.64)* | 1.01 (1.00, 1.02) | 1.15 (1.08, 1.23)* |
| 6 | 1.05 (1.03, 1.07)* | 1.53 (1.34, 1.74)* | 1.00 (1.00, 1.01) | 1.16 (1.08, 1.24)* |
| 7 | 1.05 (1.03, 1.06)* | 1.60 (1.39, 1.84)* | 1.00 (1.00, 1.01) | 1.16 (1.08, 1.25)* |
| 8 | 1.04 (1.03, 1.06)* | 1.67 (1.44, 1.93)* | 1.00 (1.00, 1.01) | 1.16 (1.08, 1.25)* |
| 9 | 1.04 (1.03, 1.06)* | 1.74 (1.49, 2.03)* | 1.00 (1.00, 1.01) | 1.16 (1.08, 1.26)* |
| 10 | 1.04 (1.03, 1.05)* | 1.80 (1.53, 2.12)* | 1.00 (0.99, 1.01) | 1.16 (1.08, 1.26)* |
| 11 | 1.04 (1.02, 1.05)* | 1.87 (1.58, 2.22)* | 1.00 (0.99, 1.01) | 1.16 (1.07, 1.26)* |
| 12 | 1.04 (1.02, 1.05)* | 1.94 (1.62, 2.32)* | 1.00 (0.99, 1.01) | 1.16 (1.07, 1.26)* |
| 13 | 1.03 (1.02, 1.05)* | 2.00 (1.66, 2.41)* | 1.00 (0.99, 1.01) | 1.16 (1.07, 1.26)* |
| 14 | 1.03 (1.02, 1.05)* | 2.06 (1.69, 2.51)* | 1.00 (0.99, 1.01) | 1.16 (1.06, 1.26)* |
| 15 | 1.03 (1.02, 1.04)* | 2.12 (1.73, 2.60)* | 1.00 (0.99, 1.01) | 1.15 (1.06, 1.26)* |
| 16 | 1.03 (1.01, 1.04)* | 2.18 (1.76, 2.70)* | 1.00 (0.99, 1.01) | 1.15 (1.05, 1.26)* |
| 17 | 1.03 (1.01, 1.04)* | 2.24 (1.79, 2.80)* | 1.00 (0.99, 1.01) | 1.15 (1.05, 1.26)* |
| 18 | 1.02 (1.01, 1.04)* | 2.29 (1.81, 2.89)* | 1.00 (0.99, 1.01) | 1.15 (1.05, 1.26)* |
| 19 | 1.02 (1.01, 1.04)* | 2.34 (1.84, 2.98)* | 1.00 (0.99, 1.01) | 1.15 (1.04, 1.26)* |
| 20 | 1.02 (1.01, 1.04)* | 2.39 (1.86, 3.08)* | 1.00 (0.99, 1.01) | 1.15 (1.04, 1.26)* |
| 21 | 1.02 (1.01, 1.03)* | 2.44 (1.88, 3.17)* | 1.00 (1.00, 1.01) | 1.15 (1.04, 1.27)* |
| 22 | 1.02 (1.01, 1.03)* | 2.49 (1.90, 3.26)* | 1.00 (1.00, 1.01) | 1.15 (1.04, 1.27)* |
| 23 | 1.02 (1.00, 1.03)* | 2.53 (1.92, 3.34)* | 1.00 (1.00, 1.01) | 1.15 (1.04, 1.27)* |
| 24 | 1.02 (1.00, 1.03)* | 2.57 (1.93, 3.43)* | 1.00 (1.00, 1.01) | 1.15 (1.04, 1.28)* |
| 25 | 1.02 (1.00, 1.03)* | 2.61 (1.94, 3.51)* | 1.00 (1.00, 1.01) | 1.16 (1.04, 1.29)* |
| 26 | 1.01 (1.00, 1.03)* | 2.65 (1.95, 3.59)* | 1.00 (1.00, 1.01) | 1.16 (1.04, 1.30)* |
| 27 | 1.01 (1.00, 1.03)* | 2.68 (1.96, 3.67)* | 1.01 (1.00, 1.02) | 1.17 (1.04, 1.31)* |
| 28 | 1.01 (0.99, 1.03)* | 2.71 (1.96, 3.75)* | 1.01 (1.00, 1.02) | 1.17 (1.04, 1.32)* |
| 29 | 1.01 (0.99, 1.03)* | 2.74 (1.96, 3.84)* | 1.01 (1.00, 1.02) | 1.18 (1.05, 1.34)* |
| 30 | 1.01 (0.99, 1.03)* | 2.77 (1.96, 3.92)* | 1.01 (0.99, 1.02) | 1.19 (1.05, 1.36)* |

“*” *P*<0.05.

**eFigure 1. Time series decomposition of DAT (a), DMaxT (b), DMinT (c), and DTD (d).**


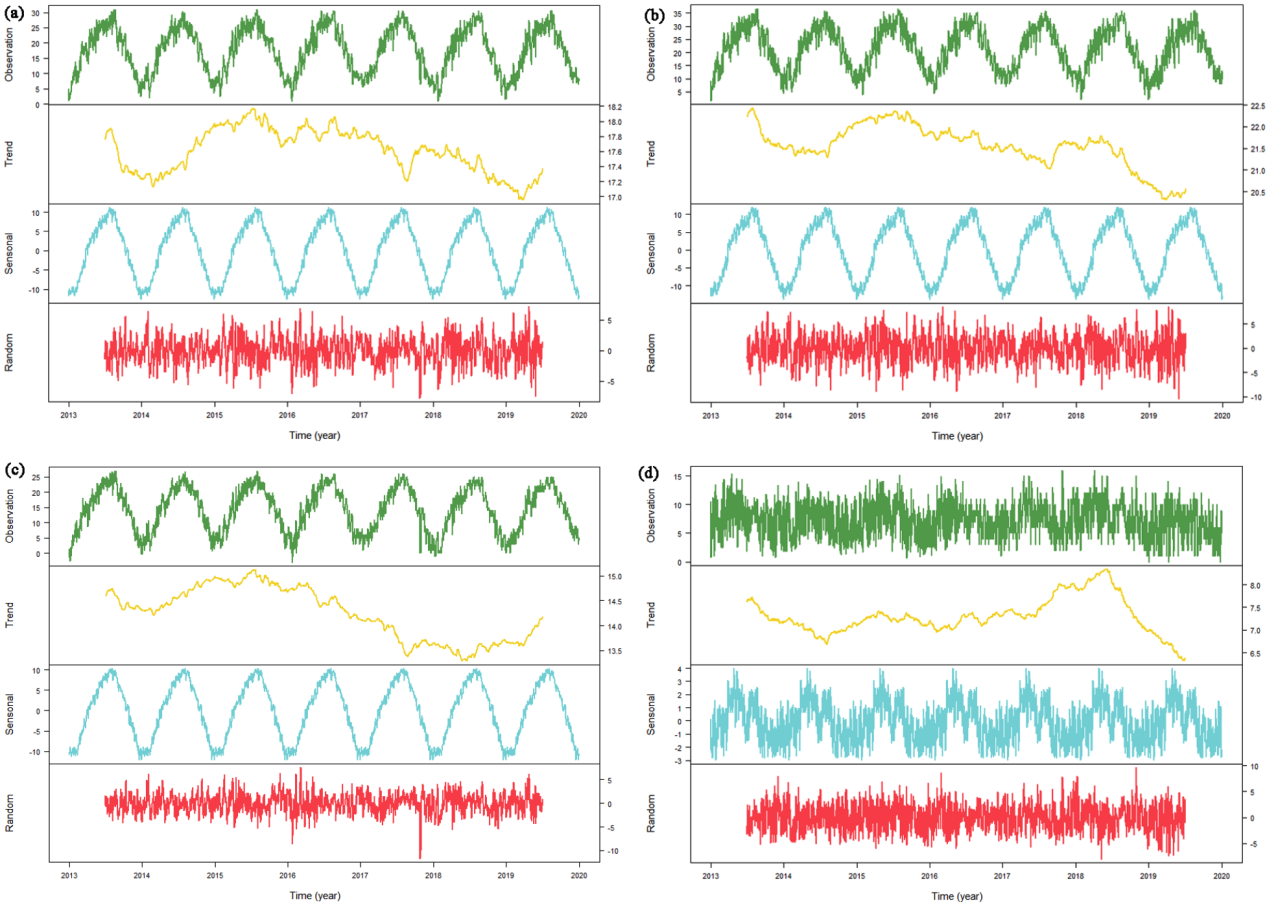


**eFigure 2. Time series decomposition of deaths from respiratory diseases (a), and circulatory diseases (b).**


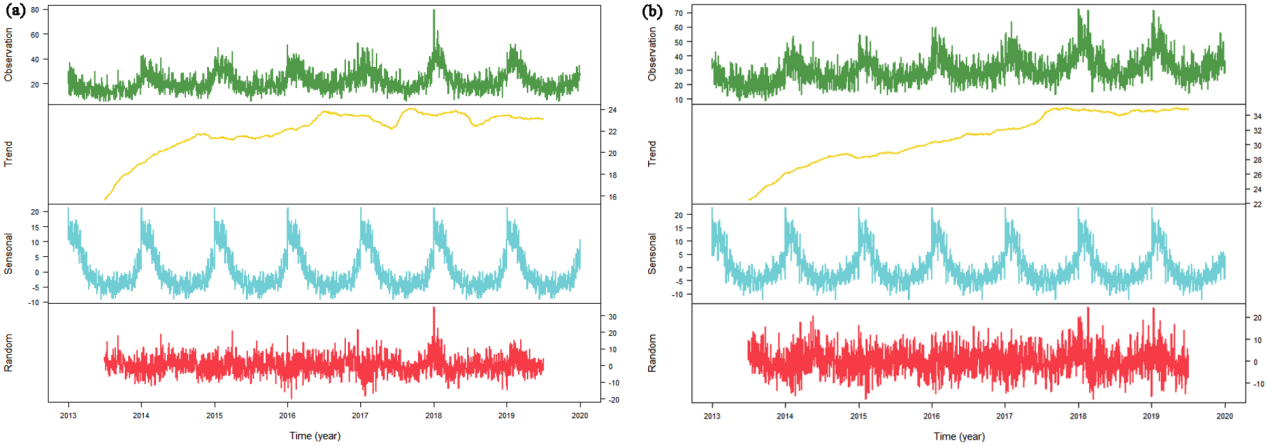


**eFigure 3. The 3-D figure of association patterns between DAT and mortality from respiratory diseases (a), circulatory diseases (b), and lag dimensions.**


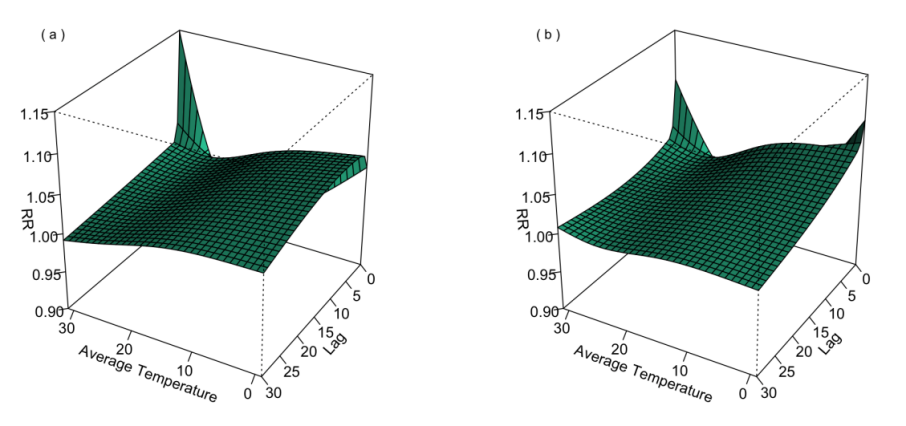


**eFigure 4. Cross-sectional data on the exposure-response relationships between DAT and mortality from all diseases.**


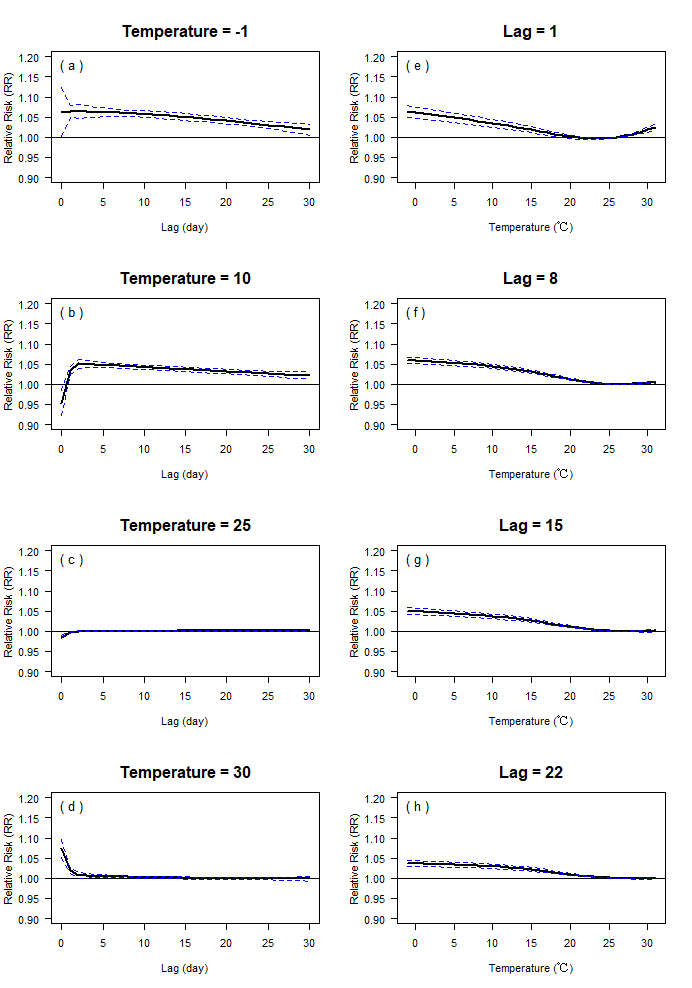

Supplement: Supplementary file 1 — Additional file 1. [file 12889_2022_13384_MOESM1_ESM.docx]
